# Disbelieved and dismissed: racial inequities in sickle cell pain management

**DOI:** 10.1590/0102-311XEN115525

**Published:** 2026-02-23

**Authors:** Victoria Simões Bernardo, Flaviene Felix Torres, Danilo Grunig Humberto da Silva

**Affiliations:** 1 Câmpus de Três Lagoas, Universidade Federal de Mato Grosso do Sul, Três Lagoas, Brasil.

Sickle cell disease is an inherited blood disorder affecting the function of red blood cells. In a typical setting, these cells should deliver oxygen to tissues and remove carbon dioxide produced during respiration ^1^. However, in sickle cell disease patients the blood cells become stiff, sticky, and shaped like sickles or crescent moons instead of being soft and round. These abnormally shaped cells can block blood flow, causing intense pain, organ damage, and a range of other serious complications ^1^ ^,^ ^2^. In Brazil, approximately 3,500 children are born each year with sickle cell disease, and recent data show a median life expectancy of only 32 years for individuals with sickle cell disease, compared to 69 years for the general Brazilian population ^3^. While a global condition, sickle cell disease disproportionately affects people of African descent and other historically marginalized populations, making it not only a medical issue but also a social justice concern. Thus, people with sickle cell disease face not only the biological consequences of their illness but also the cumulative effects of poverty, racial discrimination, and limited access to healthcare ^4^. Every time a person with sickle cell disease walks into an emergency room in pain, they carry more than just the immense physical agony of a vaso-occlusive crisis; they carry suspicion, they carry judgment. They carry a legacy of racial and medical bias so deeply rooted that their suffering is often minimized or outright ignored ^5^.

As Brazilian researchers who have spent the past ten years studying the molecular mechanisms of this condition (i.e., understanding why some individuals experience more severe symptoms than others) and searching for therapeutic alternatives to improve patients’ quality of life, we have listened to countless stories of individuals with sickle cell disease being denied pain relief, accused of drug-seeking, or simply not believed. Their pain is not seen as “real enough”, their lives not “worthy enough”. As a disclosure, we would like to make clear that the reflections presented in this paper are based on personal observations and ongoing exchanges within our research group, as well as with colleagues in the field, patients, and healthcare professionals. Despite these interactions taking place in informal settings, the discussions offered valuable insights into the lived realities of people with sickle cell disease. Additionally, we conducted a literature review using scientific databases (e.g., PubMed, Google Scholar, Web of Science, and SciELO). Searches used keywords such as sickle cell disease, racism, pain, institutional racism, and healthcare access.

The combination of this dual type of research allowed us to critically analyze how structural racism shapes the healthcare experiences of individuals living with sickle cell disease, and with that, one thing became clear: these experiences are not isolated; they are systemic. These pain episodes are not only common but can be severe and life-threatening, since they can last for hours or even days, severely affecting patients’ daily lives. Approximately 20% of individuals with sickle cell disease experience a severe pain episode at least once a month, and 10-20% of them may develop acute chest syndrome, a leading cause of death in sickle cell disease, within just three days of admission ^5^. Research also shows that individuals living with sickle cell disease often endure chronic pain that coexists with acute episodes, contributing to a diminished quality of life and higher rates of depression and anxiety. Despite this high burden of illness, patients’ pain is frequently underestimated or even completely dismissed, especially among black individuals, exposing the deep inequities in healthcare systems ^6^ ^,^ ^7^.

Racism should be understood not merely as interpersonal prejudice, but also as a structural power system that organizes and hierarchizes society, which produces social and health inequities that disproportionately affect black and Indigenous populations. Institutional racism manifests itself in Brazil through barriers to access, poor quality of care, and statistical invisibility due to insufficient collection of racial data in health information systems ^8^ ^,^ ^9^. This exclusionary structure perpetuates inequalities from the past and highlights persistent barriers to care ^9^. International data also illustrate how racism affects social determinants of health in other contexts ^10^ ^,^ ^11^. Recognizing racism as a determinant of health is therefore essential to understand sickle cell disease, where pain and suffering are often delegitimized, and to strengthen responses such as Brazilian National Comprehensive Health Policy for the Black Population (PNSIPN, acronym in Portuguese) ^12^.

Moreover, the perception of prejudice is consistently associated with worse health outcomes, reinforcing how stigma becomes embodied and produces measurable consequences. In Maranhão, one-third of patients with sickle cell disease reported experiencing prejudice, and these individuals had significantly lower quality of life scores ^13^. A growing body of research reflects this reality, and it is not unique to Brazil - similar histories are being heard worldwide. Even in highly developed countries like the United States as shown by Anderson et al. ^5^, who highlights how implicit bias leads to the under-treatment of pain in sickle cell disease, historically racialized as a “*negro blood disorder*”, especially among black patients. Similarly, Hood et al. ^7^ show that perceived racial bias and health-related stigma significantly lower the quality of life in children, particularly among older girls with the disease, which highlights intersectional vulnerabilities.

In Brazil, Carvalho et al. ^14^ and Vilela et al. ^4^ revealed how people with sickle cell disease face structural discrimination, including delayed treatments, reduced access to specialists, exclusion from clinical research, and social invisibility, compounding their physical vulnerability. Such inequities are systemic, rooted in social disparities shaped by slavery and reinforced by structural racism. Beyond the clinic, stigma also permeates family, social, and romantic relationships ^4^. Such findings align with broader evidence that racism accumulates over the life course, limiting education, employability, and healthcare access ^4^ ^,^ ^15^.

Pain is one of the universal unifiers and should unite us as humans, but for many with sickle cell disease it isolates them. The health system that should be a source of relief, often becomes a space of retraumatization. Even the language used by healthcare professionals, with phrases such as “*Are you sure it’s that bad?*” or “*You don’t look like you’re in pain*”, chips away at their dignity. Such skepticism reflects deeply ingrained bias and stigma, which accumulate over time and shape healthcare experiences. But change is possible. Community-based initiatives show that interventions grounded in dialogue and co-creation of knowledge can disrupt stigma and bias, like those discussed by Brown et al. ^16^. They promoted an in-person event called *Sickling is Not Seeking* in a large public hospital.

The initiative was presented during grand rounds to emergency and internal medicine teams led by a palliative care physician and a community advocate who lives with sickle cell disease. The event included a short video of the patient’s perspective (called *They Don’t Believe Me*), which highlighted stigmatizing language, and even an overview of the U.S. National Heart, Lung, and Blood Institute (NHLBI) sickle cell disease pain guidelines. Over 60 healthcare professionals participated in the study, and the results showed a remarkable impact of the intervention. Physicians and residents reported profound self-reflection. Some of them even acknowledged that they had previously doubted patients’ pain or used stigmatizing language in medical notes. Additionally, the video opened space for an honest dialogue, with one resident confessing she had once felt relieved when a patient left the emergency room against medical advice. Others recognized the subtle ways bias influences clinical decisions, including assumptions based on a patient’s appearance ^16^.

However, the sustainability of such interventions remains a challenge. Maintaining attitudinal change requires a thorough curricular reform in medical education and continuing professional training. Integrating patient narratives into medical and interprofessional education (i.e., engaging nurses, social workers, and psychologists) can help address the multifaceted needs of patients and ensure lasting change. Beyond direct clinical interventions, the participation of patient organizations that advocate for them, such as the Brazilian National Federation of Associations of People with Sickle Cell Disease (FENAFAL, acronym in Portuguese), is essential. The creation of FENAFAL is an important milestone in the history of sickle cell disease in Brazil, as this organization has historically denounced institutional racism, advocated for neonatal screening policies, and fought for recognition of the unique health needs of people with sickle cell disease, contributing to landmark achievements like inclusion of sickle cell disease in the Brazilian National Neonatal Screening Program, distribution of hydroxyurea through the public health system, and, more recently, mandatory notification of sickle cell disease cases in Brazil ^17^ ^,^ ^18^. These milestones illustrate how grassroots mobilization and political advocacy shape public health responses and reframe sickle cell disease as an issue of racial justice.

This shows that spaces where people with sickle cell disease are not only heard, but also lead the conversation, education of patients and professionals can occur, which is key to successful interventions. Participation in associations reduces isolation, strengthens self-esteem, and offers spaces of empowerment, highlighting that patients are not passive victims but active agents in shaping health policies and practices. These collective dimensions are crucial for counteracting stigma, shifting the narrative from individual blame to systemic accountability ^19^ ^,^ ^20^. Listening, genuinely listening, is an act of care and resistance. The health system needs to recognize that individuals with sickle cell disease hold essential lived experiences, and they should be active contributors to shaping, communicating, and implementing health knowledge. Ultimately, to address sickle cell disease is to confront not only a genetic condition but also the social and racial injustices that determine who suffers, how they are treated, and whose pain is believed.

This is why we write: because the voices of those living with sickle cell disease need to echo beyond hospital walls. Because we cannot change what we continue to ignore. And because the question should never be “*Why are you still in pain?*” but rather “*What can we do to help you heal?*”.

## Data Availability

Not applicable.
